# Idiopathic Oblique Muscle Hematoma as a Cause of Lateral Abdominal Pain

**DOI:** 10.31662/jmaj.2021-0210

**Published:** 2022-03-18

**Authors:** Kenta Takenaka, Yuki Otsuka, Takuya Nishina, Hideki Kiriyama

**Affiliations:** 1 Emergency Center, Okayama City Hospital, Okayama, Japan; 2 Department of General Medicine, Okayama University Graduate School of Medicine, Dentistry and Pharmaceutical Sciences, Okayama, Japan; 3 Department of Surgery, Okayama City Hospital, Okayama, Japan

**Keywords:** Abdominal wall hematoma, Acute abdomen, Lateral abdominal pain, Oblique muscle hematoma

A 48-year-old Japanese man was admitted to our emergency department with acute-onset sharp left-sided abdominal pain during desk work. Ureterolithiasis was initially suspected; however, it was peculiar in that no costovertebral angle tenderness was detected. Instead, he demonstrated widespread left-sided lateral abdominal tenderness with positive Carnett’s sign. Computed tomography revealed isodensity area along the left internal and external oblique muscles and transversus abdominis muscle, which was consistent with idiopathic oblique muscle hematoma (OMH) (Figure 1).

**Figure 1.**
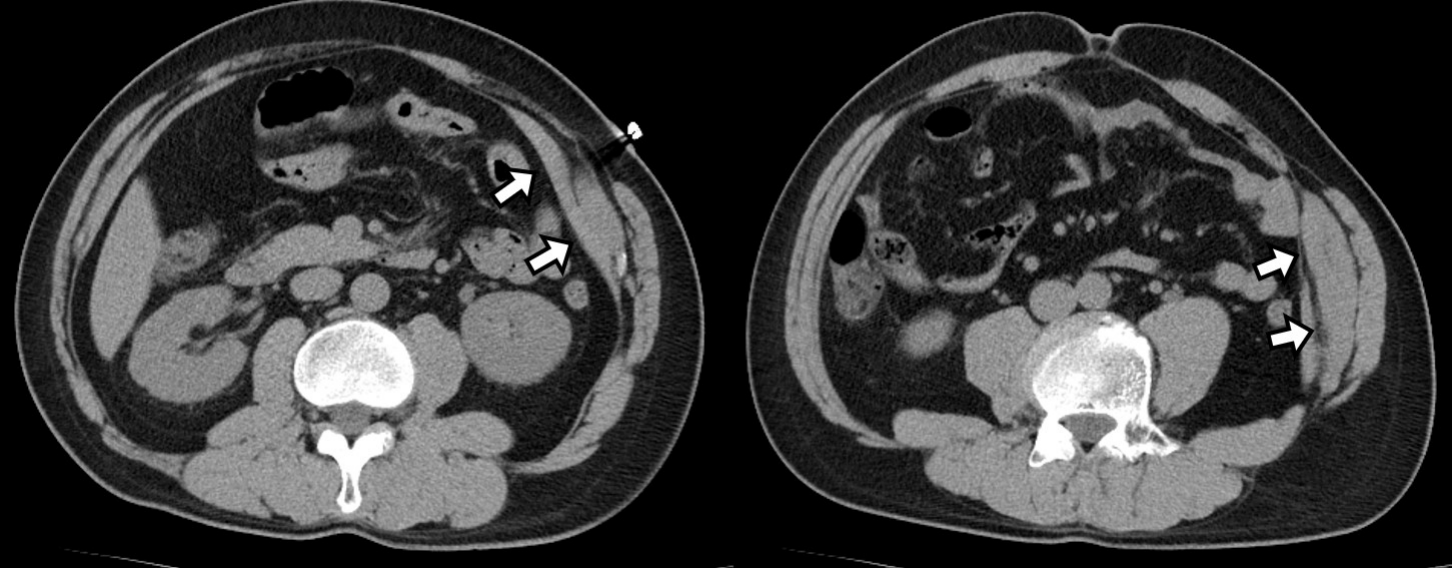
Computed tomography revealed isodensity area along the left internal and external oblique muscles and transversus abdominis muscle, suggesting oblique muscle hematoma.

Abdominal wall hematoma is a rare cause of acute abdomen, most of which are rectus sheath hematomas (RSH) ^(1)^. RSH can be misdiagnosed as intraperitoneal diseases, such as diverticulitis, peptic ulcer, or ovarian torsion ^(2)^. Similarly, we suggest that OMH, which is an extremely rare cause of abdominal wall hematoma due to the rupture of the deep iliac circumflex artery or intercostal artery, might be misdiagnosed as ureterolithiasis due to its presentation as lateral abdominal pain. About 4% are fatal ^(1)^, thus, clinicians must include OMH in their differentials.

## Article Information

### Conflicts of Interest

None

### Author Contributions

All authors contributed to patient care. Kenta Takenaka wrote the manuscript and the other authors revised it.

### Informed Consent

Written informed consent was obtained from the patient to publish.

### Approval by Institutional Review Board (IRB)

This study did not require IRB approval.

## References

[1] Shimodaira M, Kitano T, Kibata M, et al. An oblique muscle hematoma as a rare cause of severe abdominal pain: a case report. BMC Res Notes. 2013;6(1):18. 23327472 10.1186/1756-0500-6-18 PMC3552714

[2] Luhmann A, Williams EV. Rectus sheath hematoma: a series of unfortunate events. World J Surg. 2006;30(11):2050-5. 17058030 10.1007/s00268-005-0702-9

